# Spectroscopic and in silico investigation of the interaction between GH1 β‐glucosidase and ginsenoside Rb_1_

**DOI:** 10.1002/fsn3.2153

**Published:** 2021-02-09

**Authors:** Shuning Zhong, Mi Yan, Haoyang Zou, Ping Zhao, Haiqing Ye, Tiehua Zhang, Changhui Zhao

**Affiliations:** ^1^ College of Food Science and Engineering Jilin University Changchun China

**Keywords:** ginsenoside Rb_1_, interaction, molecular docking, multispectral method, β‐glucosidase

## Abstract

The function and application of β‐glucosidase attract attention nowadays. β‐glucosidase was confirmed of transforming ginsenoside Rb_1_ to rare ginsenoside, but the interaction mechanism remains not clear. In this work, β‐glucosidase from GH1 family of Paenibacillus *polymyxa* was selected, and its gene sequence *bglB* was synthesized by codon. Then, recombinant plasmid was transferred into *Escherichia coli* BL21 (DE3) and expressed. The UV–visible spectrum showed that ginsenoside Rb_1_ decreased the polarity of the corresponding structure of hydrophobic aromatic amino acids (Trp) in β‐glucosidase and increased new π‐π^*^ transition. The fluorescence quenching spectrum showed that ginsenoside Rb_1_ inhibited intrinsic fluorescence, formed static quenching, reduced the surface hydrophobicity of β‐glucosidase, and K_SV_ was 8.37 × 10^3^ L/M (298K). Circular dichroism (CD) showed that secondary structure of β‐glucosidase was changed by the binding action. Localized surface plasmon resonance (LSPR) showed that β‐glucosidase and Rb_1_ had strong binding power which KD value was 5.24 × 10^–4^ (±2.35 × 10^–5^) M. Molecular docking simulation evaluated the binding site, hydrophobic force, hydrogen bond, and key amino acids of β‐glucosidase with ginsenoside Rb_1_ in the process. Thus, this work could provide basic mechanisms of the binding and interaction between β‐glucosidase and ginsenoside Rb_1_.

## Graphical abstract

The interaction mechanism between GH1 β‐glucosidase and ginsenoside Rb1 innovatively studied by UV, fluorescence, CD, LSPR, and molecular docking. MD simulations revealed binding sites for ginsenoside Rb_1_ to bind to β‐glucosidase, the hydrophobic force, hydrogen bond, and the important amino acid residues.

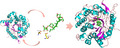

## 1 INTRODUCTION

β‐glucosidase is a general term of glycoside hydrolases that specifically catalyze the hydrolysis of oligosaccharides (usually containing 2 to 6 monosaccharide residues), alkyl and aromatic group terminal nonreducible β‐D‐glucosidase, thereby releasing monosaccharides and corresponding ligands (Lan et al., 2019). So far, β‐glucosidase is widely found in archaea (Schröder et al., 2014), bacteria (Akram et al., 2018), eukaryotes (B. Li & Renganathan, 1998), and plants (Ketudat Cairns et al., 2015), and it performs extensive and vital physiological functions. According to the consistency of the amino acid sequence, glycoside hydrolases were divided into several families, and the classification information was entered into the CAZy website for real‐time updates (http://www.cazy.org). There are up to now 133 families of glycoside hydrolases. According to this classification, β‐glucosidase is distributed in families 1, 3, 5, 9, 30, and 116 (Xia et al., 2016). Recently, research on β‐glucosidase mainly focuses on the microbial sources of β‐glucosidase, and the biochemical properties of β‐glucosidase from different species vary greatly. To determine the biochemical properties of β‐glucosidase, researchers have isolated, purified, or recombinant a large number of microbial β‐glucosidase (T.‐H. Kim et al., 2018).

Most β‐glucosidases belong to broad substrates specific β‐glucosidases, which can simultaneously hydrolyze disaccharide or oligosaccharide substrates, alkane glycoside substrates, and aromatic glycoside substrates. β‐glucosidase is widely used in industry (Ferreira et al., 2018), agriculture (Vazquez et al., 2019), food, and medicine fields. In food processing, as a part of the food flavor enzyme, β‐glucosidase can produce gentian oligosaccharide for coffee, chocolate, and other products. Because of the use for improving the flavor or taste, β‐glucosidase can digest the flavor precursors in fruits (Gueguen et al., 1996), tea (Su et al., 2010), wine (Hemingway et al., 1999), and then release the flavor. In the research and development of food and health care products, β‐glucosidase can release functional aglycones because of its hydrolytic activity to a variety of bioactive substances, such as isoflavone aglycones (Horii et al., 2009) and sapogenins (Huq et al., 2016).

Saponins are complex compounds in glycosides which composed of sapogenin and glycosyl. They are mainly distributed in many herbal medicines, such as ginseng, *Platycodon grandiflorum*, and liquorice (Zhang et al., 2020). Modern pharmacological research has found that the main active component of ginseng is ginsenoside (Attele et al., 1999). Currently, more than 100 kinds of single ginsenoside have been isolated and identified (Chen et al., 2020). Some ginsenosides with low content and high medicinal activity are called rare ginsenosides (J.‐E. Kim et al., 2019; Yang et al., 2020). At present, it has been confirmed that the ginsenoside Rb_1_, which has the highest content in ginsenoside, can be hydrolyzed by β‐glucosidase to remove the glucose unit in its chemical structure and generate rare ginsenosides with simpler structure and easier absorption by human body (M. Kim et al., 2005). The transformation of ginsenosides in vitro is mainly through the β‐glucosidase enzymatic hydrolysis of the main ginsenosides (Cui et al., 2019), resulting in different types of ginsenoside subtypes (Liu et al., 2019). Therefore, it is of great significance to study the substrate specificity or selectivity of β‐glucosidase for its effective utilization. According to the literature, although a variety of glycoside hydrolases have been reported the ability of ginsenoside transformation, such as β‐D‐glycosidase and α‐L‐arabinopyranosidase (T.‐H. Kim et al., 2018), similar studies mainly focus on the characterization of enzymatic properties and transformation properties. Nevertheless, the study of the interaction and its mechanism between glycosidase and ginsenoside molecules is rarely reported.

The correct understanding of the interaction between macromolecules and small molecules of compounds can be carried out through the mutual corroboration of multispectral experimental results and the visualization of molecular dynamics simulation. In previous studies, for the important human serum albumin (HSA) in human body, the preparation method of berberine nanoparticles (nano‐BER) was studied to improve its solubility in aqueous phase and the formation of its complex with human serum albumin (HSA) and total transferrin (HTF) (Sharifi‐Rad et al., 2020). The effect of nano‐cur binding on the interaction of hsa‐htf binary system and ternary system was studied by multi spectral and molecular dynamics simulation (Mokaberi et al., 2020). In addition, the interaction between hemoglobin (HB) and lomefloxacin (LMF) was also determined by fluorescence spectroscopy, and the molecular simulation results were used as evidence (Mokaberi et al., 2019). DNA is the main target in organism and participates in important intercellular processes. Small molecules can bind with histone DNA and damage the division, growth, inhibition, and apoptosis of cancer cells. The interaction between histone H1 calf thymus DNA (CT‐DNA) complex and propyl acridone (PA) was studied using multispectral, viscosity, and molecular simulation techniques (Shakibapour et al., 2019). As an important class of proteins, enzymes play an important catalytic role in biochemical reactions. The effects of three silver nanoparticles with different particle sizes on the binding of curcumin with lysozyme under physiological conditions were studied by spectroscopic and zeta potential techniques (Kamshad et al., 2019).

Given the above, this work will consider the interaction between ginsenoside Rb_1_ and β‐glucosidase. For Paenibacillus *polymyxa* with ginsenoside transformation ability, we synthesized its β‐glucosidase gene which was the key to the catalytic process and then constructed it on the pET‐28a (+) vector. The recombinant expression vector pET‐28a (+)‐*bglB* was transformed into *E*. *coli* BL21 (DE3) for expression in order to obtain high purity β‐glucosidase.

In this work, we reported the interaction between ginsenoside Rb_1_ and β‐glucosidase by spectroscopic method such as UV, fluorescence, and CD and evaluated the effect of Rb_1_ on the conformation of β‐glucosidase. LSPR would be used to determine the specific binding force of their interaction. For it is difficult to achieve the binding conformation in the micro state by conventional experimental means, molecular docking will provide visually simulate the best binding site of β‐glucosidase and ginsenoside Rb_1_ and expected to reveal the important hydrogen bond force and important amino acid residues in the interaction. We aimed to explain or give some insights into the interaction mechanism of β‐glucosidase and ginsenoside Rb_1_.

## 2 MATERIALS AND METHODS

### 2.1 Strains, vectors, and reagents

*Escherichia coli* DH5α was preserved in our laboratory for gene cloning and large amplification vector plasmids and recombinant plasmids and used as the cloning host. *E. coli* BL21 (DE3) was preserved in our laboratory and served as the expression host. Plasmids containing the target gene were synthesized and connected to the prokaryotic expression vector pET‐28a (+) for protein expression by Sangon biological engineering (Shanghai, China) Co., LTD. All restriction endonucleases and ligases were purchased from Takara (Dalian, China). Isopropyl‐β‐D‐thiogalactoside (IPTG) was purchased from Dingguo Changsheng Biotechnology Co. Ltd (Beijing, China). Kanamycin sulfate was purchased from Shanghai Macklin Biochemical Co., Ltd. Ni‐nitrilotriaceticacid (Ni‐NTA) agarose and Ni‐NTA column was purchased from Qiagen (Hilden, Germany). Ginsenosides Rb_1_ was purchased from Shanghai Yuanye Co., Ltd. All other chemical and reagents were purchased from Sangon Co., Ltd (Shanghai, China) unless being indicated otherwise.

### 2.2 Design and synthesis of β‐glucosidase gene

Using NCBI to query β‐glucosidase from Paenibacillus *polymyxa*, which belongs to the first family of glycoside hydrolases (GenBank: M60211.1), the size of the β‐glucosidase gene was 1,344 bp, the amino acid sequence was 448 aa, and its molecular weight was predicted to be 52 kDa. In view of its origin in bacteria, it can be expressed in *E*. *coli*. In order to insert the target gene into the expression vector, restriction enzyme sites were introduced into the 5' and 3' ends of the foreign gene, respectively. At the same time, the amino acid sequence encoded by the original β‐glucosidase gene was not changed. The optimized gene is named *bglB*. The gene sequence was entrusted to Sangon Biotechnology Co. Ltd. to complete the whole gene synthesis. The fragment was digested with NdeI and XhoI and then cloned into similarly digested plasmid pET‐28a (+). The plasmid DNA was extracted using the Wizard® Plus SV Minipreps DNA Purification System (Promega). After the synthesis, the whole gene was sequenced to ensure the fidelity of the target gene.

### 2.3 Expression and purification of recombinant β‐glucosidase

The 2.0 ml engineered bacteria *E*. *coli* BL21(DE3) were added into 100.0 ml Luria‐Bertani (LB) liquid medium containing 30 μg/mL kanamycin and oscillated for 12 hr at 37 ℃ at 180 r/min. Then, the medium was transferred to 2.0 L LB liquid medium containing 30 μg/ml kanamycin and oscillated the culture at 37°C and 180 r/min until the absorbance value (OD_600_) measured at the wavelength of 600 nm reached 0.60–0.80. Later, 1 M IPTG was added to the culture group until the final concentration was 0.5 mM and induced overnight at 30°C, 120 r/min. The fermentation broth was centrifuged at 4°C at 8,000 r/min for 5 min; then, supernatant was discarded and the bacteria cell were collected. Weighing about 2.5 g bacteria cell, then added 25 ml disodium hydrogen phosphate–citric acid buffer (20 mM, pH = 7.0) and resuspensed. At the end, the concentration of bacteria was 0.1 g/mL, the ultrasonic crushing was conducted under ice bath for 60 min (working time for 3 s, interval time for 3 s), and the separation was conducted at 4°C at 8,000 r/min. After 60 min, the supernatant was collected as a crude enzyme solution. Since six His‐tags had been added to the *bglB* gene sequence, the target protein can be obtained after being washed by eluent on a nickel column after protein expression. Nickel column affinity chromatography was used for purification and removed the heteroprotein by eluate containing 20 mM imidazole. The target protein was collected with 150 mM imidazole eluent and dialyzed overnight in 20 mM pH = 7.0 disodium hydrogen phosphate–citric acid buffer. Sodium dodecyl sulfate–polyacrylamide gel electrophoresis (SDS‐PAGE) was performed to verify protein purity. The protein was then concentrated, freeze‐dried, and stored at −20 ℃ for future use.

### 2.4 Absorption spectrum of β‐glucosidase and ginsenoside Rb_1_

The UV spectrum of the β‐glucosidase and ginsenoside Rb_1_ mixture samples was obtained using a UV Visible (UV‐Vis) spectrometer (Cary series, Agilent Technologies). The UV‐Vis absorption study of 10 μM β‐glucosidase was performed in 50 mM phosphate buffer at pH 7.0 to reach the final ginsenoside Rb_1_ concentrations from 0–100 μM. Put the two universal UV cuvettes into the UV spectrometer, and the constant temperature is 298 K. One was a β‐glucosidase solution containing 3 ml as the control group, and the other was the experimental group with a 3 ml buffer. The parameters were as follows: spectral scanning speed 400 nm/s; wavelength range 200–400 nm. At 298 K, the absorption changes of the complex were evaluated at 280 nm. The contribution of β‐glucosidase was subtracted from β‐glucosidase and ginsenoside Rb_1_ mixture to compare the contribution of different concentrations of ginsenoside Rb_1_ to the spectrum.

### 2.5 Fluorescence spectrum of β‐glucosidase and ginsenoside Rb_1_

The fluorescence spectrum of the mixture of β‐glucosidase and ginsenoside Rb_1_ was obtained by a fluorescence spectrophotometer (Hitachi f‐7000). The instrument was connected to a constant temperature control tank to characterize the molecular interaction at 298 K, 304K, and 310K. The formation of the complex was estimated using the fluorescence quenching method described above, with some minor modifications. At the same time, the excitation wavelength of excitation and emission was set to 280 nm, and the slit width was 5 nm. In the range of 300 to 500 nm, the emission spectrum was collected to increase the equal part of 100 μM ginsenoside Rb_1_ solution to the fixed initial volume (2.0 ml) of 10 μM β‐glucosidase solution to prepare a series of solutions. Stern–Volmer equation can be used to study the quenching mechanism.

(1) $$F_{0}/F=1+K_{q}\tau_{0}\left[Q\right]=1+K_{\text{sv}}\left[Q\right]$$

In the process of fluorescence quenching type analysis, an important and unavoidable problem was the fluorescence internal filtering effect (Kamshad et al., 2019). The internal filtering effect would affect the quenching data, which made the calculated quenching constant have an error. Therefore, the fluorescence intensity used in the research process is corrected by the following formula.

(2) $$F_{\text{cor}}=F_{\text{obs}}\times 10^{(\text{Aex + Aem})/2}$$

*F*_cor_ was the corrected fluorescence intensity; *F*_obs_ was the uncorrected fluorescence intensity; A_EX_ is the UV‐Vis absorption value of the quencher at the excitation wavelength; A_EM_ is the UV‐Vis absorption value of the quencher at the emission wavelength.

### 2.6 Nonradiative energy transfer of β‐glucosidase and ginsenoside Rb_1_

10 μM β‐glucosidase was accurately prepared. The fluorescence spectrum of β‐glucosidase was obtained by scanning in the range of 280 nm to 600 nm with the excitation wavelength of 280 nm by the fluorescence spectrophotometer. The concentration of 10 μM of ginsenoside Rb_1_ was accurately prepared, and the UV–visible spectrum was determined in the range of 200 nm ~ 430nm. The integral area of the overlapped peak of the characteristic fluorescence spectrum of β‐glucosidase with that of the characteristic ultraviolet absorption spectrum of Rb_1_ was obtained by MATLAB program. Then, the R‐value was calculated by the formula of binding distance, and the binding distance between β‐glucosidase and ginsenoside Rb_1_ was obtained. The calculation formula of the combined distance is as follows.

(3) $$E=\frac{R_{0}^{6}}{R_{0}^{6}+r^{6}}=1‐\frac{F}{F_{0}}$$

### 2.7 Circular dichroism (CD) spectrum of β‐glucosidase and ginsenoside Rb_1_

To discover the effects of binding on the secondary structure of β‐glucosidase induced by Rb_1_, circular dichroism (CD) spectra (200–260 nm) of 10 μM β‐glucosidase were collected on a MOS‐500 Circular Dichroism Spectrometer (Bio‐Logic Science Instruments, Grenoble, France). The optimal concentrations of ginsenoside Rb_1_ were confirmed through the preliminary experiment which is 100 μM in 50 mM phosphate buffer at pH 7. In the presence or absence of ginsenoside Rb_1_, both samples were evaluated with a 1 mm cell, under constant nitrogen flush at 298 K. The scan rate was 200 nm × min^−1^, the response time was 1 s, and the bandwidth was 1 nm. The changes in the percentage of secondary structure elements of β‐glucosidase were computed by using CDNN software.

### 2.8 Localized surface plasmon resonance (LSPR) of β‐glucosidase and ginsenoside Rb_1_

To determine the kinetics of the interaction between β‐glucosidase and ginsenoside Rb_1_, we used the LSPR method (open SPR XT, Nicoya Life sciences, on, Canada). COOH chip was installed according to the standard operation procedure of the open sprtm instrument. The first step was to start to run PBS buffer (pH 7.4) at the maximum flow rate (150 μl/min). After reaching the signal baseline, 200 μl of 80% IPA (isopropanol) was injected for 10 s to discharge bubbles. After reaching the baseline was to wash the sample ring with buffer solution and empty it with air. After the signal reached the baseline, adjusted the buffer flow rate to 20 μl/min. Added 200 μl EDC / NHS solution to wash the sample ring with buffer solution, and made it empty with air. The purified β‐glucosidase was diluted to 2 μM and added into the activation buffer to fix the protein on the COOH sensor chip. All experiments were conducted with filtered and degassed PBS buffer (NaCl 137 mM, KCl 2.7 mM, Na_2_HPO_4_ 4.3 mM, and KH_2_PO_4_ 1.4 mM) at a continuous flow rate of 20 μl/min at 20°C. The analyte which concentrations of ginsenoside Rb_1_ were 0.5 to2 μM was immobilized by β‐glucosidase. The surface of the sensor chip will be regenerated by flowing buffer for a long time. The association and dissociation phases of 240 s and 600 s were recorded, respectively. Data analysis included a dissociation curve of up to 400 s. The analyte was passed through the blank COOH sensor chip to measure the background response of ginsenoside Rb_1_ combined with the sensor chip. The analysis software used for the results of this experiment was trace tracer (Ridgeview Instruments AB, Sweden), and the analysis method was one to one analysis model.

### 2.9 Molecular docking simulation

The crystal structure of β‐glucosidase was studied by molecular docking and downloaded from the protein database (www.rcsb.org, PDB: 2Z1S). The initial structure of ginsenoside Rb_1_ was modeled by a molecular simulation package Sybyl 7.3, and Tripos force field and Gasteiger‐Marsili were used to charge optimizes molecular geometry. The main modeling steps were as follows: (a) Using blast or PSI‐BLAST to search the template of the target sequence. (b) Using structure alignment method to compare and overlap the template. (c) Using sequence alignment method to compare the target sequence with the sequence of the template structure. (d) Using modeler to generate the model of target sequence. Procheck program was applied to the structure after modeling to get the Ramachandran diagram. The molecular docking of β‐glucosidase and ginsenoside Rb_1_ were carried out with AUTODOCK Vina software. Lamarckian (LGA) genetic algorithm was used to calculate the possible conformation of ginsenoside Rb_1_ molecule bound to protein *bglB*. In the docking process, at most 10 conformations of the compound were considered, and the conformation with the lowest binding free energy was taken for further analysis.

### 2.10 Statistical analysis

Data were repeated at least thrice and expressed as the mean values ± standard deviations (*SD*). The Tukey test was used to identify significant differences between means (*p* <  0.05) utilizing a one‐way ANOVA test. Typical spectra and data were presented as figures.

## 3 RESULTS

### 3.1 Gene synthesis and expression vector construction of β‐glucosidase

**FIGURE 1:**
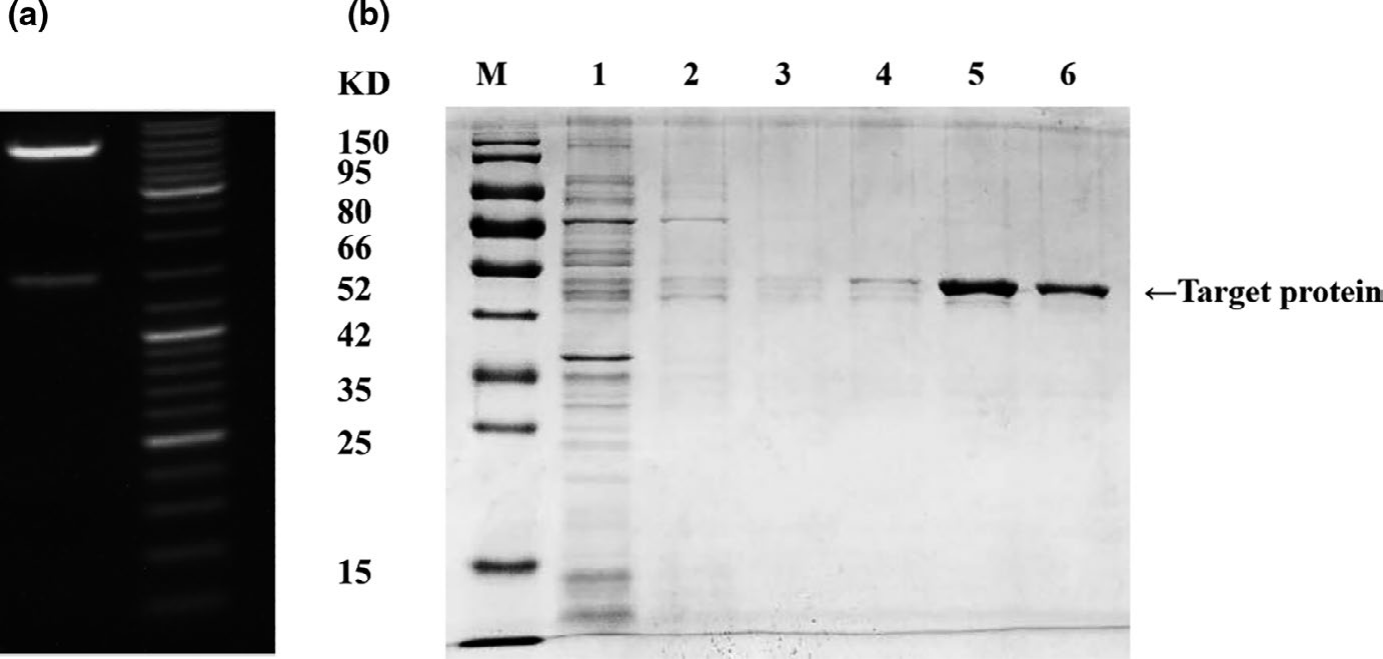
DNA and protein electrophoresis. (a) Enzyme digestion analysis of pET‐28a (+) ‐bglB (Enzyme:Ndel/Xhol;Expected size:1,356 + 5.3 KB. (b) SDS‐PAGE analysis for column purified β‐glucosidase. Column M represents protein marker, and columns 1 to 6 from left to right represent β‐glucosidase samples after elution with imidazole eluent at the concentration of 0, 20, 50, 100, 150, and 200 mM until a single band being obtained

The recombinant vector pET‐28a (+)‐*bglB* was identified by agarose gel electrophoresis. According to the Figure 1a, the recombinant vector PET‐28a (+)‐*bglB* was verified by double digestion of recombinant vector by agarose gel electrophoresis. The size of the 1,356 + 5.3K bp was PET‐28a (+)‐*bglB*, which was neat and bright on the edge. Therefore, it can be preliminarily confirmed that the recombinant plasmid has been successfully constructed. The correct recombinant plasmid pET‐28a (+)‐*bglB* was introduced into the receptive cells for transformation, and the required monoclonal was screened after overnight culture in LB plate.

### 3.2 Expression and purification of β‐glucosidase

The β‐glucosidase gene of *bglB* was PCR amplified, then cloned into the pET‐28a (+), and transformed in *E*. *coli* BL21 (DE3) for the expression of target enzyme. In the presence of IPTG, large amounts of a protein were produced, at a molecular mass of 52 kDa, corresponding to that expected for the full‐length protein encoded by *bglB*. When whole *E*. *coli* cells were lysed in a buffer solution, the β‐glucosidase was soluble and catalytically active. As shown in Figure 1b, purification of β‐glucosidase was achieved by Ni‐NTA Agarose and a single band protein identified by SDS‐PAGE can be obtained. Therefore, on the one hand, it could be determined that under the condition of 30°C, 200 rpm oscillation induction, the desired inducible expression product, that is, prokaryotic expression of target protein β‐glucosidase, can be better obtained in the precipitate induced overnight. On the other hand, it can be determined that the gene fragment size of β‐glucosidase induced by prokaryotic expression is 52 kDa. The predicted expression molecular weight of *bglB* was consistent with other research result from the Paenibacillus *polymyxa* (Huang et al., 2019).

### 3.3 UV‐Vis absorption studies of β‐glucosidase and ginsenoside Rb_1_

**FIGURE 2:**
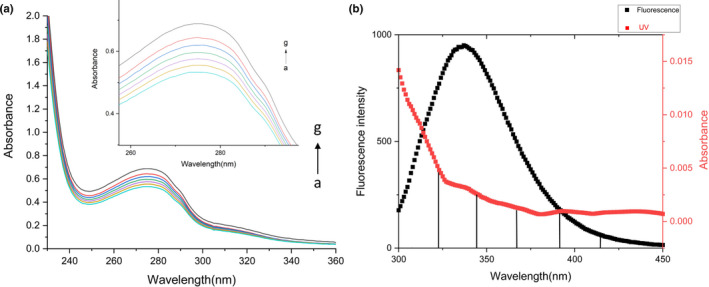
(a) The UV absorption spectra of various concentrations ginsenoside Rb_1_ (0, 10, 20, 40, 60, 80, 100 μM, from the curve a to g) and β‐glucosidase (10 μM) (b) Emission spectra of β‐glucosidase and excitation spectra with ginsenoside Rb_1_ UV absorption spectra

UV‐Vis absorption spectrophotometry is regarded as an effective method to study the structural change of target protein. There are two important absorption bands of β‐glucosidase: (a) the absorption band at about 280 nm is composed of aromatic amino acids such as tryptophan, tyrosine, and phenylalanine. Under the action of the enzyme, the absorption peak of β‐glucosidase is negatively related to the activity of the enzyme. (b) Soret absorption band at 405 nm. The absorption peak at about 280 nm is composed of benzene heterocyclic structure of aromatic amino acid residues. Therefore, we can analyze and judge the interaction between ginsenoside Rb_1_ and β‐glucosidase protein solution according to the change of the UV absorption peak intensity and the displacement of the maximum absorption peak. Due to the benzene ring structure of Rb_1_ containing UV absorption peak, it is necessary to add the ginsenoside Rb_1_ of equal concentration on both sides of the experimental group and the blank group. The Figure 2a showed the influence of ginsenoside Rb_1_ on the UV‐Vis absorption spectrum of β‐glucosidase. According to the change of absorption spectrum of β‐glucosidase, it could be inferred that ginsenoside Rb_1_ bound to β‐glucosidase and changed its conformation with the protein. It could be seen that the absorption intensity of β‐glucosidase at about 280 nm raised with the increase of ginsenoside Rb_1_ concentration, indicating that Rb_1_ caused the hydrophobic groups of aromatic amino acids (Trp) in β‐glucosidase to be more wrapped and the polarity of corresponding structure to be weakened (Agrawal et al., 2016). In addition, this process led to the formation of a new conjugation system between β‐glucosidase and small drug molecules, and the addition of a new π‐π^*^ transition. The energy of the π‐π^*^ transition increased, resulting in a blue shift of the absorption peak at about 280 nm and the formation of a drug protein ground state complex. The ginsenoside Rb_1_ is a major panaxadiol from ginseng root, and it can gradually remove the glucose groups at C3 and C20 sites by glycosidase catalysis to generate secondary ginsenosides (Tian et al., 2016).

The shift and intensity of UV absorption peak are related to the surrounding environment. The absorption peak of β‐glucosidase at 280 nm was mainly caused by the π‐π^*^ transition of aromatic heterocycles in aromatic amino acids. With the addition of ginsenoside Rb_1_, the intensity of the absorption peak at 280 nm was enhanced, which indicated the interaction between ginsenoside and β‐glucosidase. The addition of the small molecular compound led to the extension of peptide chain in protein molecule, which exposed the tryptophan residues in the subdomain and increased the hydrophilicity. It was confirmed that ginsenoside Rb_1_ formed a complex with β‐glucosidase. However, the microenvironment was not significantly changed by observing the UV Vis absorption spectrum of the complex.

### 3.4 Fluorescence quenching studies of β‐glucosidase by ginsenoside Rb_1_

**FIGURE 3:**
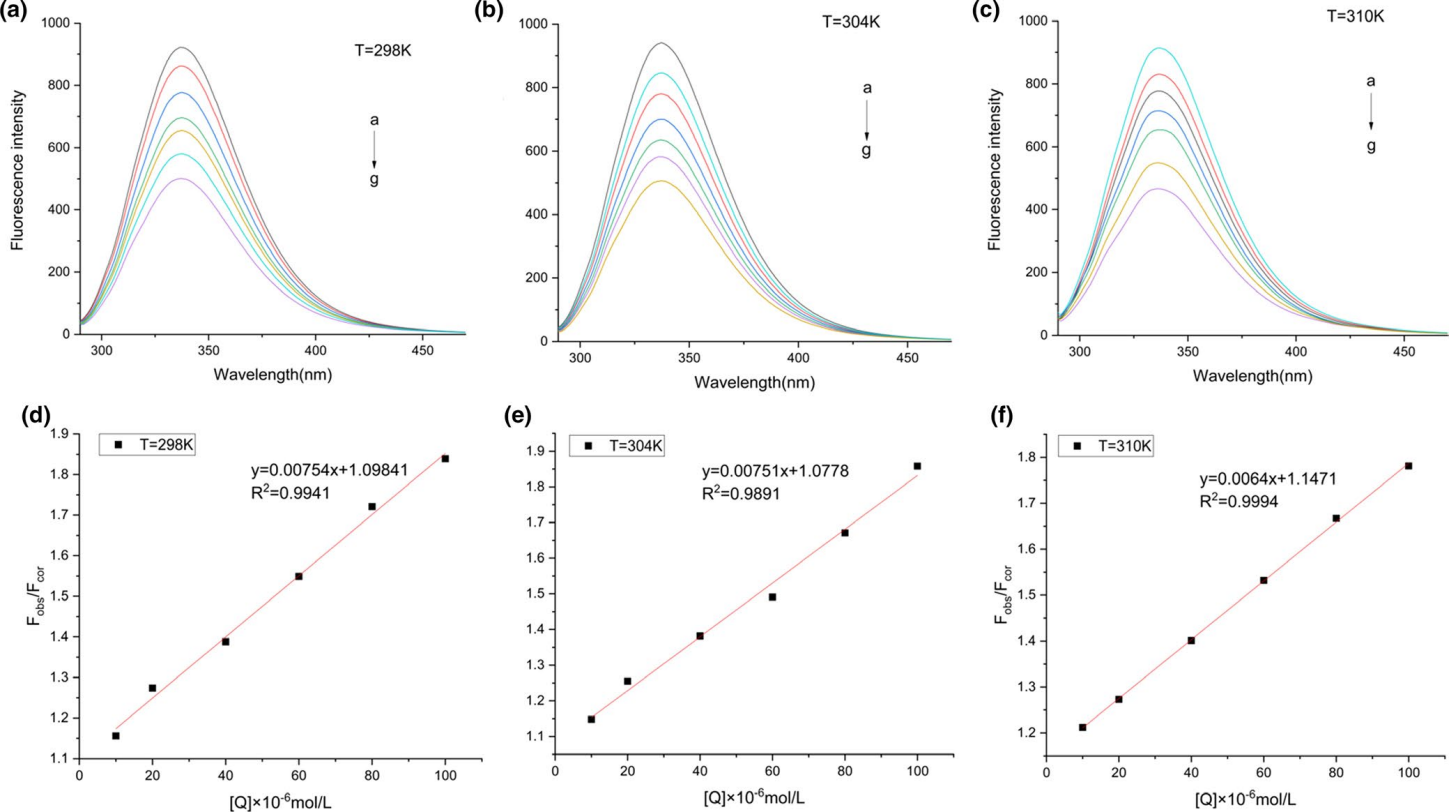
Effect of ginsenoside Rb_1_ on the fluorescence spectra of β‐glucosidase. (a‐c) The fluorescence quenching spectra of β‐glucosidase (10 μM) as affected by upward concentrations of ginsenoside Rb_1_ (0, 10, 20, 40,60, 80, 100 μM from top to bottom) at 298K, 304K, and 310K. (d‐f) The Stern–Volmer plot for the fluorescence quenching of β‐glucosidase by ginsenoside Rb_1_ at 298K, 304K, and 310K

The binding mechanism of ginsenoside Rb_1_ and β‐glucosidase was further studied by fluorescence experiment. Fluorescence quenching is a common tool to study the interaction between ligands and proteins as well as substrate and enzyme (L. Li et al., 2016). It provides valuable information about quenching mechanism, binding sites, and binding constants (Günther et al., 2018). Fluorescence quenching refers to the reduction of fluorescence intensity, which may lead to collision quenching, energy transfer, formation of ground state complexes, molecular rearrangement, and other process of many other types of molecular interactions(Wang et al., 2020). The intrinsic fluorescence of β‐glucosidase was from three aromatic amino acid residues (tyrosine, tryptophan, and phenylalanine) (Luo et al., 2019). In the fluorescence study, when the excitation wavelength was set to 280nm, the emission fluorescence of β‐glucosidase was mainly attributed to its intrinsic fluorescence residues Tyr and Trp, while when the excitation wavelength was set to 295nm, it minimized the emission of Tyr residues, which selectively excites Trp residues. The quenching mechanism of ginsenoside Rb_1_ on β‐glucosidase and other binding parameters were studied by using the excitation wavelength of 280nm. At 298K, the maximum fluorescence emission intensity of natural enzyme was 349 nm. As shown in the Figure 3a, different concentrations of ginsenoside Rb_1_ (0, 10, 20, 40, 60, 80, and 100 μM) quenched β‐glucosidase at 298K in a phosphate buffer of 50mM pH of 7.0. The measurements were also made at 304 K and 310 K at the same concentration conditions as shown in Figure 3b and Figure 3c. With the increase of ginsenoside Rb_1_ concentration, the fluorescence intensity of β‐glucosidase decreased. A red shift was observed with the increase of ginsenoside Rb_1_ concentration at the excitation wavelength of 280nm. These results indicate that the microenvironment of tyrosine, tryptophan, and phenylalanine residues in β‐glucosidase has changed. Therefore, ginsenoside Rb_1_ had a significant effect on the conformation of enzyme, and its quenching mechanism was usually divided into static quenching mechanism and dynamic quenching mechanism. The higher the temperature led to the faster diffusion, and more collisions happened. Therefore, the dynamic quenching constant would increase with the increase of temperature. As shown in Figure 3d, in order to determine the fluorescence quenching mechanism, the fluorescence quenching data were analyzed according to the known Stern–Volmer equation. A linear nature of the Stern–Volmer plot (y = 0.00754x + 1.0984, R^2^ = 0.9941) was found in the Rb_1_ interaction with β‐glucosidase. It could be seen that ginsenoside Rb_1_ had a good linear relationship between *F*_0_/*F* and [Q] at 298K. *K*_SV_ values of Rb_1_, as the quenching constant *K*_SV_ value is 8.37 × 10^3^ L/M, and the rate constant *K*_q_ value is 8.37 × 10^11^ L/M·s. In the Stern–Volmer plot, F_0_ was the maximum fluorescence intensity when β‐glucosidase solution was without dropping anything, and F was the maximum corrected fluorescence intensity when β‐glucosidase solution was added with ginsenoside Rb_1_. *K*_SV_ was Stern–Volmer quenching constant. [Q] was the molality concentration of ginsenoside Rb_1_. *K*_q_ was the rate constant of bimolecular quenching process. τ0 was the fluorescence lifetime of the biomacromolecule, which was about 10^‐8^s. Therefore, the *K*_SV_ and *K*_q_ values differ by 10^–8^ times.

The experiment was carried out at three temperatures at 298K, 304K, and 310K, respectively. The data were shown in the Table 1. The *K*_SV_ of the interaction between ginsenoside Rb_1_ and β‐glucosidase increased with the increase of temperature, and *K*_q_ was greater than the dynamic quenching constant of 2.0 × 10^10^ L M^‐1^s^‐1^. It can be preliminarily inferred that the fluorescence quenching of β‐glucosidase by Rb_1_ belongs to static quenching (Kayukawa et al., 2019).

### 3.5 Binding distance of ginsenoside Rb_1_ and β‐glucosidase

Energy transfer can be divided into radiation energy transfer and nonenergy transfer, among which nonradiation energy transfer is also called fluorescence resonance energy transfer (FRET) (Hemachandran et al., 2017). If there is radiation energy transfer between donor and acceptor, the fluorescence spectrum of the fluorescent material will be deformed, and the fluorescence intensity of the fluorescent material will be quenched. The main chromophores of β‐glucosidase are tryptophan (Trp) and tyrosine (Tyr). The fluorescence intensity and the maximum emission peak shift of this residue can directly show the microenvironment changes of excellent amino acid and tyrosine residues. It can be seen from the Figure 2b that β‐glucosidase had a strong fluorescence intensity at the excitation wavelength of 280 nm and had a large degree of overlap with the UV absorption spectrum of Rb_1_. The main light‐emitting group of β‐glucosidase was tryptophan residue, and when the UV absorption spectrum of Rb_1_ was at the binding position of β‐glucosidase, the binding distance *r* is less than 7 nm, and the tryptophan residue of β‐glucosidase can emit fluorescence. The binding distance *r* of Rb_1_ to β‐glucosidase was 1.79 nm, and the binding distance of Rb_1_ to β‐glucosidase was less than 7 nm. Therefore, the nonradiative energy transfer between Rb_1_ and β‐glucosidase can be preliminarily determined. In conclusion, the energy of β‐glucosidase was transferred to Rb_1_, and the fluorescence intensity of β‐glucosidase on tryptophan residue was reduced, and then, fluorescence quenching occurred.

### 3.6 Conformation changes of β‐glucosidase induced by ginsenoside Rb_1_

**FIGURE 4:**
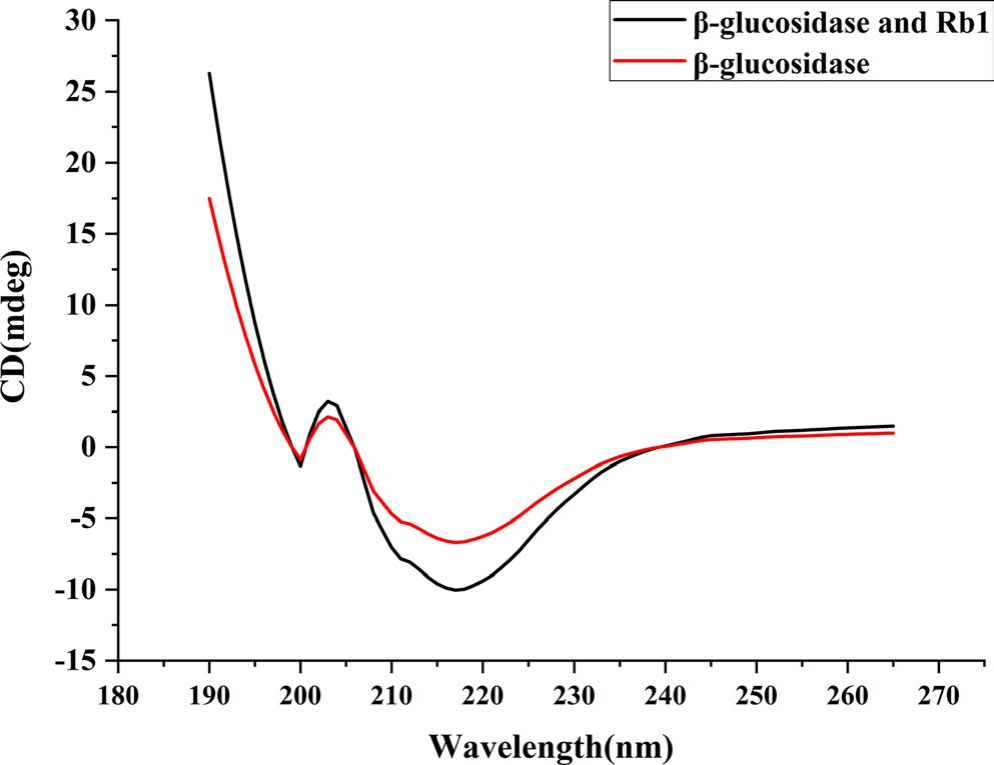
The CD spectra of β‐glucosidase secondary structures upon binding to ginsenoside Rb_1_ (0 and 100 μΜ) at 25°C

CD spectroscopy is used to be a convenient and precise technique, and it has been widely used to screen the changes in protein conformation based on these characteristics (Bhagyalekshmi et al., 2019). The secondary structure of the target protein was monitored by CD spectroscopy (Jahromi et al., 2020), and the effect of ginsenoside Rb_1_ on the secondary structure of β‐glucosidase was analyzed. 0 and 100 μM Ginsenoside Rb_1_ and 10 μM β‐glucosidase were incubated in 50 mM pH 7.0 and 298 K phosphate buffer for 3 min. The CD of the samples was recorded in the range of 190‐260nm. As shown in the Figure 4, the spectrum of β‐glucosidase showed two negative bands at 198 and 218 nm, which are the characteristics of α helix and α helix / random helix in β‐glucosidase structure. With the addition of 100 μM ginsenoside Rb_1_, the CD intensity of β‐glucosidase at 198 and 218 nm decreased significantly, which indicated that the secondary structure of β‐glucosidase changed significantly with the increase of α‐helix content. The content of different secondary structure of β‐glucosidase was calculated by CDNN program. The secondary structure of β‐glucosidase includes 10.3% β‐sheet, 36.8% α‐helix, 14.3% β‐bend, and 38.6% random coil. With the increase of ginsenoside Rb_1_ concentration, the content of α‐helix increased, while the content of β‐sheet decreased, and random coil did not change significantly. According to these results, we think that the structure of β‐glucosidase was stable by increasing the content of α‐helix. In addition, the decrease of β‐sheet structure indicated that ginsenoside Rb_1_ had an important interaction with hydrophobic contact when compared with the same type of research (Matsuo & Gekko, 2019). The circular dichroism diagram of β‐glucosidase had a positive peak near 204nm, and an obvious negative peak near 218nm, which was the characteristic peak of the α‐helix in the protein. With the addition of ginsenoside Rb_1_, the strength of positive and negative peak increased, indicating that the content of α‐helix increased continuously. The formation of this phenomenon was consistent with the change trend of enzyme activity, suggesting that α‐helix a necessary structure to maintain the conformation of the active center of enzyme molecules. After the ginsenoside Rb_1_ interacting with β‐glucosidase, the hydrogen bonding occurred with polar amino acid residues in protein molecules, which improved the stability of the complex.

### 3.7 Calculation of Binding Parameters between β‐Glucosidase and ginsenoside Rb_1_

**FIGURE 5:**
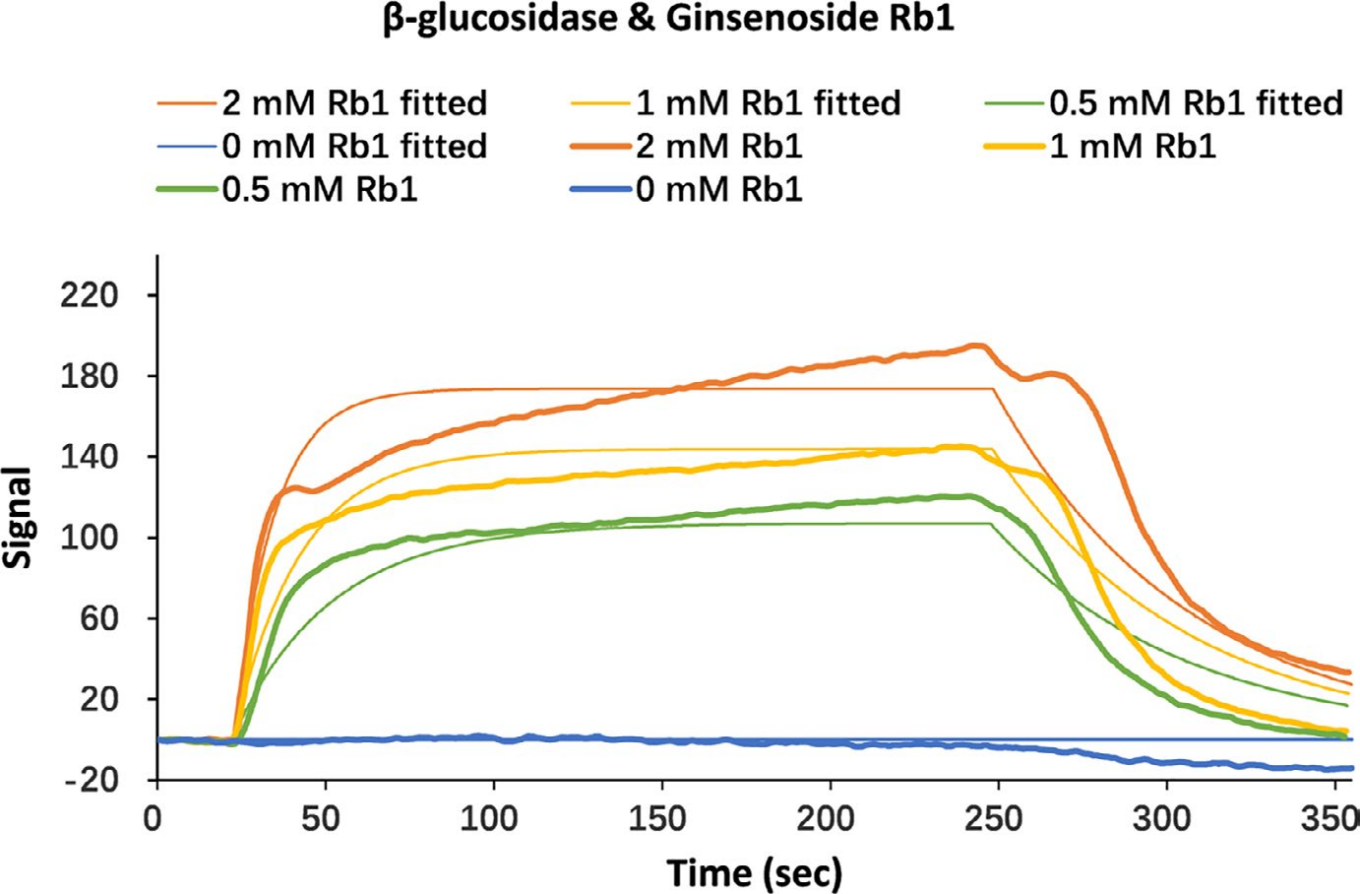
Local surface plasmon resonance (LSPR) of the interaction between β‐glucosidase and ginsenoside Rb_1_. The representative curves of the ginsenoside Rb_1_ concentrations (blue, 0 mM; green, 0.5 mM; yellow, 1 mM; orange, 2 mM;) show the association and dissociation phases of the interaction between β‐glucosidase and ginsenoside Rb_1_. Thin lines of the same color represent the fit between the data and the 1:1 binding model

Localized surface plasmon resonance (LSPR) is an optical phenomenon, which can be used to track the interaction between biomolecules in natural state in real time (Elsawy et al., 2016; P. Li et al., 2020). This method has no damage to biological molecules and does not need any markers. We further confirmed the interaction between β‐glucosidase and ginsenoside Rb_1_ by LSPR. As shown in Figure 5, LSPR data clearly showed that β‐glucosidase interacted with ginsenoside Rb_1_ to form a stable 1:1 complex, and the equilibrium dissociation constant (KD) was 5.24 × 10^–4^ (±2.35 × 10^–5^)M. LSPR data also showed a high correlation the dissociation rate of this interaction, the Ka was 29.7 (±6.62 × 10^2^ /M × s) and the Kd was 1.56 × 10^–2^ (±2.17 × 10^–5^) /s .

### 3.8 Molecular Docking analysis

The molecular docking analysis of β‐glucosidase and its substrate was carried out by using AUTODOCK Vina program. Except for the size of docking box, all docking parameters were default and chose the conformation with the best affinity (i.e., the lowest affinity value, which was −8.9kcal/M in this docking case) to be selected as the docking conformation for subsequent molecular dynamics simulation. The binding energy of molecular docking was slightly higher than that of spectral experiments, possibly because molecular docking was conducted in a simulated vacuum environment, while spectral experiments were conducted in a solvent environment, which was consistent with other studies that have been reported(Dehghani Sani et al., 2018; Shakibapour et al., 2019).

**FIGURE 6:**
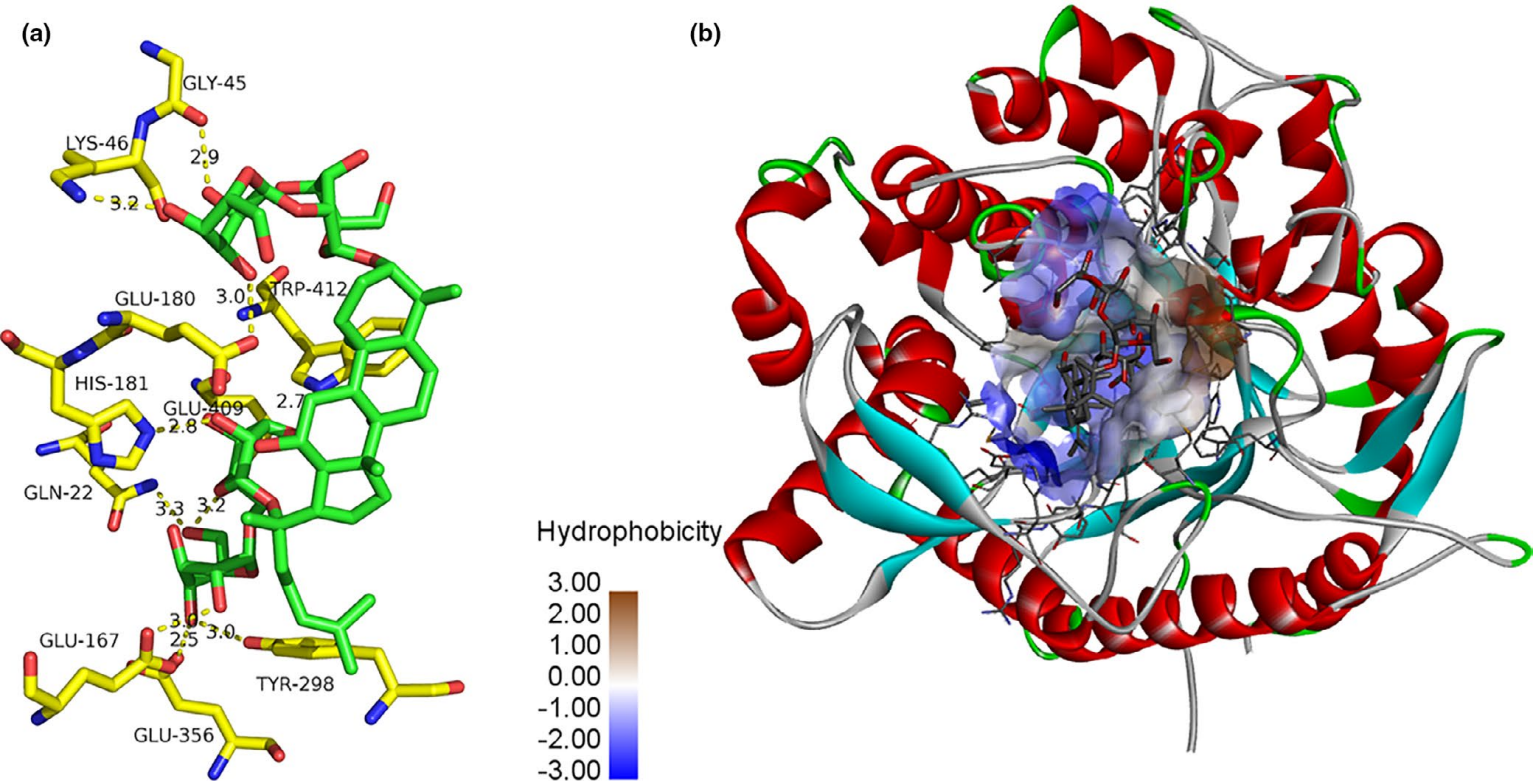
The binding modes obtained from molecular docking (MD). (a) MD simulation results showing that the hydroxyl group of ginsenoside Rb_1_ and several active pocket residues of β‐glucosidase all formed hydrogen bond interaction and the amino acid residues Glu167 and Glu356 are very important for the catalysis of β‐glucosidase. (b) MD simulation results showing possible binding sites of ginsenoside Rb_1_ on β‐glucosidase. The hydrophilicity of β‐glucosidase binding pocket had a binding effect on ginsenoside Rb_1_

As shown in Figure 6b, it could be seen from the docking results that the substrate binding site located in a barrel structure formed by β‐sheet around the glycosidase center. However, the ginsenoside Rb_1_ was not completely in the barrel, because its structure was relatively extended, most of the groups were in the upper part of the barrel structure and interacted with multiple loop structures of receptor protein. The loop structures were also important for the combination of substrates. In addition, because the binding site was shallow and not into the barrel structure, it had certain hydrophilicity. The glycoside substrate had many hydroxyl groups and strong hydrophilicity. Therefore, the hydrophilicity of the binding pocket was also important for substrate binding, which conformed to the basic principle of energy matching. It could be inferred that there were many hydrogen bonds between the glycoside substrate and the receptor protein to maintain their binding. Due to its special extension, one part of the substrate was bound to protein β‐sheet, and the other part was exposed to solvent environment. As shown in the Figure 6a,a hydrogen bond interaction was formed between the substrate hydroxyl and a plurality of active pocket residues. These residues included Gly45 (skeleton oxygen), Lys46 (side chain amino group), Glu180 (side chain carboxyl group), His181 (side chain N), Gln22 (side chain amide group), Glu167 (side chain carboxyl group), Tyr298 (side chain hydroxyl group), and Glu356 (side chain carboxyl group). These hydrogen bonds were essential for the binding of substrates and further catalysis. Therefore, these residues could also be regarded as hot spots which affect the substrate binding, and their functions could be further verified by point mutation in subsequent experiments. As mentioned in the literature, Glu167 and Glu356 were essential for the catalysis of glycosidases (Zhou et al., 2019). The substrate involved in this study had a relatively extended spatial conformation, which led to a large steric hindrance in the process of entering the active pocket. This made the distance between glycoside bond (oxygen atom) and catalytic residue larger. The distance between Glu167 and its carboxyl group is 6.1 Å. To some extent, the occurrence of catalysis was limited. In the later stage, in order to improve the enzyme activity, the substrate could be mutated to combine with the pocket residue, so that the distance would be reduced. Based on the understanding of the sequence and structure of β‐glucosidase, it could be possible to improve the activity of β‐glucosidase by site directed mutation in the future, as to realize the efficient catalysis of ginsenosides.

## 4 DISCUSSION AND CONCLUSION

In order to obtain the enzyme protein with clear gene sequence, in this study, β‐glucosidase gene sequence was optimized and synthesized by codon. The total length of *bglB* gene was 1,344 bp. With pET‐28a (+) as the expression vector, the recombinant vector pET‐28a (+)‐*bglB* was constructed and transferred into *E*. *coli* BL21 (DE3). After induction, the target protein was obtained and purified by Ni‐NTA column. The molecular weight and purity of the target protein were identified. The purified β‐glucosidase was identified as a single band by SDS‐PAGE. The obtained high purity enzyme could be used for subsequent assays to explore its activity and binding effect.

The mechanism of interaction between β‐glucosidase and ginsenoside Rb_1_ was studied by a series means of spectroscopy. With the increase of ginsenoside Rb_1_ concentration, the UV spectrum showed that there was a slight blue shift (279–277 nm) which indicated that the ginsenoside Rb_1_ caused the hydrophobic group of aromatic amino acid (Trp) in β‐glucosidase to be more wrapped. Thus, the polarity of corresponding structure was weakened and became a new π‐π* transition, so that the complex of substrate and enzyme was formed. The fluorescence quenching spectra showed that ginsenoside Rb_1_ significantly inhibited the intrinsic fluorescence of β‐glucosidase, which indicated that there was a strong interdependence between the two molecules, which was consistent with the previous UV spectrum results and the quenching type was the static quenching mechanism. The decrease of the surface hydrophobicity of β‐glucosidase indicated that the Rb_1_ bound to the hydrophobic groups on the protein surface. The circular dichroism spectrum showed that the binding of ginsenoside Rb_1_ to β‐glucosidase resulted in the change of enzyme conformation. LSPR results showed that β‐glucosidase and ginsenoside Rb_1_ had strong binding power, and the specific KD value was determined.

In the molecular docking analysis, the molar stoichiometry of β‐glucosidase and ginsenoside Rb_1_ complex (1:1) showed that it was composed of a ginsenoside Rb_1_ molecule and each β‐glucosidase protein molecule. Molecular models provided valuable insight into the nature of functional groups and forces involved in the combination, as well as the formation of visible complexes. Through the results of molecular docking, the hydrophobic force and hydrogen bond were evaluated, and the important amino acid residues to the protein in the process of β‐glucosidase catalyzing ginsenoside were found in the simulation.

In general, we systematically and innovatively studied the binding interaction of β‐glucosidase and ginsenoside Rb_1_ by combining various spectroscopic methods, LSPR and molecular docking. Those results provided a new idea for the gaps in research of ginsenosides and glycosidases which only involve in biotransformation findings in the past. Thus, our findings consist a substantial and definite addition to the present understanding of the interaction between ginsenoside Rb_1_ and β‐glucosidase from GH1 family. It also provided a research method basis for the binding mechanism of enzymes and their atypical substrates in the catalytic process.

## 5 INFORMED CONSENT

Informed consent was obtained from all individual participants included in the study.

## CONFLICT OF INTEREST

The authors declare that they have no conflict of interest.

## AUTHORS CONTRIBUTIONS

**Shuning Zhong** involved in methodology development or design of methodology, writing—original draft preparation, software programming, and software development. **Mi Yan** involved in investigation. **Haoyang Zou** involved in data curation. **Ping Zhao** involved in validation verification. **Haiqing Ye** involved in supervision and visualization. **Changhui Zhao** involved in writing—reviewing and editing. Tiehua Zhang* involved in conceptualization ideas, resources, and project administration.

## ETHICAL STATEMENTS

This article does not contain any studies with human or animal subjects.

## ETHICAL APPROVAL

This article does not contain any studies with animals or human participants performed by any of the authors.

6

**TABLE 1:** Quenching constants and linear equations of interaction between ginsenoside Rb_1_ and β‐glucosidase at different temperatures

Agent	T(K)	Equations	*R* ^2^	*K*_SV_ (L·mol^−1^)	*K*_q_ (L·mol^−1^·s^−1^)
Ginsenoside Rb_1_	298	y = 0.00754x + 1.0984	0.9941	8.37 × 10^3^	8.37 × 10^11^
	304	y = 0.00741x + 1.0778	0.9891	8.45 × 10^3^	8.45 × 10^11^
	310	y = 0.00640x + 1.1471	0.9994	8.58 × 10^3^	8.58 × 10^11^
